# Efficacy of Rectal Systemic Administration of Mesenchymal Stem Cells to Injury Sites via the CXCL12/CXCR4 Axis to Promote Regeneration in a Rabbit Skeletal Muscle Injury Model

**DOI:** 10.3390/cells12131729

**Published:** 2023-06-27

**Authors:** Toru Ichiseki, Miyako Shimasaki, Shusuke Ueda, Hiroaki Hirata, Daisuke Souma, Norio Kawahara, Yoshimichi Ueda

**Affiliations:** 1 Department of Orthopaedic Surgery, Kanazawa Medical University, Daigaku 1-1, Uchinada-machi, Kahoku 920-0293, Japan; adeu221@kanazawa-med.ac.jp (S.U.);; 2 Department of Pathology 2, Kanazawa Medical University, Daigaku 1-1, Uchinada-machi, Kahoku 920-0293, Japan; miya0807@kanazawa-med.ac.jp; 3 Department of Pathology, Keiju Medical Center, 94, Tomioka-machi, Nanao 926-0816, Japan

**Keywords:** mesenchymal stem cells (MSCs), C-X-C chemokine ligand 12 (CXCL12)/C-X-C chemokine receptor-4 (CXCR4) axis, cardiotoxin (CTX), myosin heavy polypeptide 3 (Myh3)

## Abstract

Mesenchymal stem cells (MSCs) have been transplanted directly into lesions or injected intravenously. The administration of MSCs using these delivery methods requires specialized knowledge, techniques, and facilities. Here, we describe intrarectal systemic administration of MSCs, a simple, non-invasive route for homing to the injury sites to promote the regeneration of skeletal muscle injuries. Using a cardiotoxin (CTX)-induced rabbit skeletal muscle injury model, homing to the site of muscle injury was confirmed by intrarectal administration of MSCs; the time required for homing after intrarectal administration was approximately 5 days. In addition, the C-X-C chemokine ligand 12 (CXCL12)/C-X-C chemokine receptor-4 (CXCR4) axis was found to be involved in the homing process. Histopathological examinations showed that skeletal muscle regeneration was promoted in the MSCs-administered group compared to the CTX-only group. Myosin heavy polypeptide 3 (Myh3) expression, an indicator of early muscle regeneration, was detected earlier in the intrarectal MSCs group compared to the CTX-only group. These findings indicate that intrarectal administration of MSCs is effective in homing to the injured area, where they promote injury repair. Since intrarectal administration is a simple and non-invasive delivery route, these findings may be valuable in future research on stem cell therapy.

## 1. Introduction

Skeletal muscles are tissues in which regeneration is actively performed. Muscle satellite cells (skeletal muscle tissue stem cells) are known to play a central role in the regeneration of injured skeletal muscle tissues [1,2]. When the skeletal muscle is injured, satellite cells are activated and proliferate, either fusing with the injury site to repair damaged muscle cells or fusing to form new muscle cells [3]. Therefore, the activation of muscle satellite cells has been shown to be an important step in the regeneration of injured skeletal muscle tissues. However, age-related decreases in the number of muscle satellite cells have been reported [1], as well as decreases in self-replication functions [4], and proliferative capacity [5]. Moreover, satellite cell abnormalities are related to the pathology of Duchenne muscular dystrophy (DMD) in mdx mice, an animal model of DMD [6].

Following pathologic or mechanical damage, there is upregulation of cytokines, such as the insulin-like growth factor, which aids muscle repair [7]. C-X-C chemokine ligand 12 (CXCL12), a stromal cell-derived factor (SDF)-1, is one of these molecules known to be upregulated in time of muscle damage [8]. CXCL12 and its receptor, C-X-C chemokine receptor-4 (CXCR4), are recognized to be essential in the regulation of various biological processes involving cell motility, chemotactic response, cell adhesion, gene transcription, cell proliferation, and the survival of progenitor stem cells of the musculoskeletal system [9,10,11,12,13]. Muscle progenitor cells, which express CXCR4, have been shown to migrate toward SDF-1 during the generation of new myofibers in limb muscles [14,15]. The CXCL12/CXCR4 axis has been reported to play an important role in satellite cells responsible for muscle repair after injury [16]. 

Bone marrow mesenchymal stem cells (MSCs) possess remarkable cell therapy efficacies [17,18,19]. MSCs and Platelet-Derived Growth Factor Receptor α (PDGFRα)-positive mesenchymal progenitor cells have been shown to activate muscle satellite cells [20] and promote regeneration after various skeletal muscle injuries [21,22,23,24,25]. The positive effects of MSCs and PDGFRα-positive mesenchymal progenitor cells are reportedly due to the secretion of growth factors and their ability to differentiate into skeletal muscle cells [26,27,28]. Intramuscular administration of MSCs has also been shown to activate mechanistic/mammalian target of rapamycin complex 1 (mTORC1), which is important for maintaining and improving skeletal muscle mass [29]. Given these findings, MSCs could affect skeletal muscle disorders.

MSCs have been reported to perform tissue repair through paracrine effects [18,30,31,32], and because systemically administered MSCs exhibit strong paracrine activity, effective homing of MSCs to injury sites is an important step in MSCs therapy. Most systemically administered MSCs travel to the lungs and home to the site of injury by migrating in response to inflammatory mediators [33,34]. Systemic administration of MSCs is expected to be minimally invasive and engraftable in the affected muscle because of the homing ability of MSCs from the circulatory system to the injured muscle [35]. Moreover, intravenous administration of MSCs has been shown to inhibit high-fat, diet-induced skeletal muscle atrophy [36]. Recently, MSCs also have CXCR4, and the CXCL12/CXCR4 axis has been shown to be involved in homing to the injured area [37,38,39,40].

Generally, MSCs are directly transplanted into lesions or injected intravenously, following which they home to inflammatory or injured areas [16,17,18]. The administration of MSCs using these delivery methods requires specialized knowledge, techniques, and facilities. However, many patients have difficulty getting to the hospital; therefore, it would be beneficial to establish a route of MSCs administration that can be done at home. For example, muscular dystrophy and age-related skeletal muscle disorders are pathological conditions that can make traveling to a hospital difficult. Moreover, medications that can be taken at home will likely reduce the burden on patients and medical staff, leading to lower medical costs. Oral or intrarectal administration is the most common route of administration available to patients at home. However, oral administration (in which the therapeutic is exposed to gastric acid) might not be a realistic at-home administration method for viable MSCs.

Recently, MSCs have been rectally administered for the treatment of inflammatory bowel disease [41]. However, no reports exist on the intrarectal systemic administration of MSCs concerning homing to injury or inflammation sites.

We hypothesized that the intrarectal administration of MSCs is a simple and non-invasive delivery route through which MSCs can home to the injury site via the CXCL12/CXCR4 axis and promote muscle regeneration. To test this hypothesis, using a rabbit model of cardiotoxin (CTX)-induced muscle injury, we had the following specific objectives:

- To confirm the absorption and homing of Green Fluorescent Protein (GFP)-labeled MSCs to the injury sites after intrarectal administration by immunofluorescence (IF) staining and Western blotting (WB).
- To evaluate the effect of MSCs homing on muscle regeneration by histopathology and immunohistochemical (IHC) examination for myosin heavy polypeptide 3 (Myh3), a marker of early muscle differentiation.
- To elucidate the role of the CXCL12/CXCR4 axis in MSCs homing by knocking down CXCR4 expression in MSCs using siRNA and comparing the homing with control MSCs.

This study could provide novel insights into the mechanisms and regulation of MSCs migration and differentiation for skeletal muscle regeneration, and could have a significant impact on the field of stem cell therapy and regenerative medicine, especially for skeletal muscle diseases.

## 2. Materials and Methods

### 2.1. Experimental Animals

A total of 46 eight-week-old male Japanese white rabbits (Sankyo Labo Service, Tokyo, Japan) were used in this study. The rabbits were maintained at the Laboratory Animal Center of Kanazawa Medical University. Each experimental animal was housed in a separate cage under standard laboratory conditions (temperature: 24 °C, light/dark cycle: 12-h/12-h) and provided with food and water ad libitum. The experimental protocol for this study was approved by the Animal Research Committee of Kanazawa Medical University (#2022-15).

### 2.2. Cell Culture and GFP-Labeling of MSCs

Rabbit MSCs (DS Pharma Biomedical, Osaka, Japan and Cyagen, Silicon Valley, CA, USA) were maintained as a subconfluent monolayer culture in MSC growth medium (Cyagen, DS Pharma Biomedical) at 37 °C under 5% CO_2_. The medium was exchanged every 3 days. Passaging was routinely performed when the culture reached 70% confluence. The rabbit MSCs were transfected with pCAG-GFP (Addgene, Watertown, MA, USA), encoding GFP, using the Effectene Transfection Reagent (QIAGEN, Venlo, The Netherlands). The transfected GFP-labeled rabbit MSCs (GFP-MSCs) were harvested when the cultures reached 80% confluence [42].

### 2.3. Establishing the Model and MSCs Administration

A skeletal muscle injury model was created by injecting 10 μmol (1 mL) of CTX (Latoxan, Valence, France) into the right vastus lateralis femoris of the rabbit; CTX is widely used in skeletal muscle repair and regeneration experiments because it induces immediate inflammation and injury [43,44,45]. To confirm MSCs homing, a solution containing GFP-MSCs in an MSCs growth medium (1.0 × 10^7^ cells, 1 mL) was administered intrarectally through the anus, using a 24-gauge catheter one day after CTX injection (after muscle injury and inflammation set in). In order to prevent the leakage of administered MSCs, the anus was sealed tightly with Steri-Strip tape for 1 h after the intrarectal administration.

First, we confirmed the absorption of intrarectally administered MSCs into the rectal tissue. The two experimental groups consisted of rabbits treated with CTX and administered GFP-MSCs (intrarectal administration group [IAG]; *n* = 3) and untreated rabbits (total control group, rabbits not treated with CTX and to which MSCs were not administered, [TC]; *n* = 3). IAG rabbits were sacrificed 10 min after GFP-MSC administration and rectal tissues were promptly harvested to confirm the absorption of GFP-MSCs. Rectal tissues from TC rabbits served as the control. 

Next, to confirm homing to the site of muscle injury, IAG rabbits were sacrificed 1, 3, 5, and 7 days after GFP-MSCs administration and the right vastus lateralis was harvested (*n* = 5, each). The groups at these time points were CTX 2 days/MSCs 1 day, CTX 4 days/MSCs 3 days, CTX 6 days/MSCs 5 days, and CTX 8 days/MSCs 7 days. The left vastus lateralis muscle of the femur to which CTX was not administered was the control for MSCs homing (non-CTX group). 

Additionally, the regeneration of skeletal muscle was compared with that in the MSCs administration group by administering only CTX to the same site and sacrificing the animals after 2, 4, 6, and 8 days to collect the right vastus lateralis muscle (*n* = 3, each). The CTX-only groups were CTX 2 days, CTX 4 days, CTX 6 days, and CTX 8 days. In addition, to evaluate the effect of MSCs administration on promoting skeletal muscle regeneration, rabbits were prepared, in which only CTX was injected into the right vastus lateralis (non-MSCs administration). These rabbits were sacrificed 2, 4, 6, and 8 days after CTX administration, and the right vastus lateralis was harvested. The groups were designated as CTX 2 days, CTX 4 days, CTX 6 days, and CTX 8 days, respectively (*n* = 3, each). The right vastus lateralis of untreated rabbits (using the TC group in the rectal study, *n* = 3) was used as a control group. 

In all experiments, rabbits were sacrificed by rapid injection of sodium thiopental (Nipro Pharma, Osaka, Japan) via an auricular vein. After collection, the samples were immediately snap-frozen in liquid nitrogen for WB analysis and cryopreserved in OCT (Sakura Finetek Japan Co., Tokyo, Japan) at −80 °C for IF staining. Additionally, a part of each collected sample was immersed in formalin for histopathological and IHC examination.

### 2.4. Immunofluorescence Staining

Frozen skeletal muscle and rectal tissue sections were fixed in 4% paraformaldehyde, washed in phosphate-buffered saline (PBS), and permeabilized with 0.3% Triton X-100 in PBS. Nonspecific binding was blocked by incubating sections with 10% (*w*/*v*) bovine serum albumin (BSA; Dako Cytomation, Santa Clara, CA, USA) in PBS for 15 min. The slices were subsequently incubated with anti-GFP (Abcam, Cambridge, UK), anti-CXCL12 (Proteintech, Rosemont, IL, USA), and anti-CXCR4 (Novus Biologicals, Centennial, CO, USA) for 2 h at a concentration of 5.0, 1.0, or 1.0 µg/mL. Next, the sections were incubated with fluorescence-labeled secondary antibody; for anti-GFP, Alexa 488 (Thermo Fisher Scientific, Waltham, MA, USA) was used; and for anti-CXCL12 and anti-CXCR4, Alexa 594 was used. Next, the sections were incubated with 4′,6-diamidino-2-phenylindole (DAPI) for 30 min. After washing, the stained slices were mounted using the ProLong Diamond Antifade Mountant (Thermo Fisher Scientific). Images were taken using BZ-X700 (Keyence, Tokyo, Japan).

### 2.5. Western Blotting 

For the quantification of GFP expression, immunoblotting was performed on rabbit skeletal muscles and rectum tissues. Protein was extracted using a protein extraction solution (PRO-PREP, iNtRON Biotechnology, Seongnam, Republic of Korea). Equal amounts of protein (20 µg) were electrophoresed on a 10% polyacrylamide gel and transferred to a nitrocellulose membrane (Wako, Tokyo, Japan). The membranes were incubated with anti-GFP (1.0 µg/mL; Cell Signaling, Danvers, MA, USA) overnight at 4 °C. After incubation with peroxidase-labeled, goat anti-mouse or anti-rabbit secondary antibodies (0.7 µg/mL; Dako Cytomation) for 1 h at room temperature and vigorous washing; the nitrocellulose membrane was incubated with Chemiluminescence Luminol Reagent (Immuno Star LD, Fujifilm Wako, Japan) and photographed digitally using Image Quant LAS 4000 mini (GE healthcare Japan Co., Tokyo, Japan). Immunoblotting using the anti-actin monoclonal antibody (Sigma Chemical Co., St. Louis, MO, USA) was used for standardization. The intensity was measured using the Multi Gauge v3.1 (Fujifilm, Tokyo, Japan). Experiments were repeated at least three times.

### 2.6. Cell Culture and Transfection with Stealth siRNA against CXCR4 

Rabbit MSCs were cultured as before. At about 60% confluence, MSCs were transfected with the stealth siRNA duplex oligoribonucleotides (Invitrogen Life Technologies, Carlsbad, CA, USA). The sequence of the stealth siRNA duplex oligoribonucleotides against CXCR4 is 5′- GCCCUCAAGACUACGGUCAUCCUUA -3′, and its corresponding complementary strand is 5′- UAAGGAUGACCGUAGUCUUGAGGGC -3′ (Invitrogen Life Technologies). A negative stealth siRNA sequence was used as a control. The CXCR4 siRNA was transfected transiently using LipofectamineTM RNAiMAX (Invitrogen Life Technologies) according to the manufacturer’s instructions. Briefly, siRNA plasmid (150 pmol) was diluted in 245 µL of Opti-MEMI (Gibco, Waltham, MA, USA) and the solution was gently mixed. Lipofectamine™ RNAiMAX (Invitrogen Life Technologies) was gently mixed and 4 µL of the reagent was then diluted in 246 µL of Opti-MEMI. Then, the diluted siRNA and the diluted LipofectamineTM RNAiMAX were combined for 20 min at room temperature to allow the formation of transfection complexes. The final volume of these solutions was 0.5 mL, and the final concentration of RNA was 40 nM. The complexes were then added to each well containing cells in six-well plates while they were in the quiescent state, and all were swirled gently to ensure uniform distribution. After incubation at 37 °C for 48 h, IF staining and WB were performed.

### 2.7. Confirmation of Homing Suppression by CXCR4 Knockdown in MSCs Expressing GFP (siCXCR4/GFP-MSCs)

To confirm the involvement of the CXCL12/CXCR4 axis, 1.0 × 10^7^ siCXCR4/GFP-MSCs were administered intrarectally through the anus 1 day after CTX administration. Since this protocol showed a significant increase in homing at the muscle injury site 5 days after MSCs administration, we evaluated the area of muscle injury 5 days after siCXCR4/GFP-MSCs administration (*n* = 4) and compared the results with the newly created GFP-MSCs 5-day group (*n* = 4).

### 2.8. Histopathology and Immunohistochemistry

For histological evaluations, the rabbit femoral skeletal muscle was removed and fixed in 10% buffered formalin for 24 h. Next, the sample was decalcified by immersion in 5% formic acid for 48 h, after which, paraffin-embedded specimens were prepared. The prepared paraffin block was sliced at a thickness of 3 µm and examined using hematoxylin and eosin (H & E) staining.

To investigate the skeletal muscle regenerative effects of MSCs homing in vivo in a CTX-induced rabbit skeletal muscle injury model, immunohistochemical studies were performed using Myh3, which is expressed during the early stages of skeletal muscle regeneration [46,47].

For all groups, skeletal muscle sections were prepared. Sections obtained from the femoral proximal medial diaphysis were deparaffinized with xylene and ethanol. For antigen retrieval, the sections were autoclaved at 121 °C for 15 min. Endogenous peroxidase was eliminated using 0.3% H_2_O_2_ and blocking was performed using mouse or goat normal serum. Finally, the sections were incubated with the anti-Myh3 mouse monoclonal antibody (5.0 µg/mL; Santa Cruz Biotechnologies, Dallas, TX, USA) at 4 °C overnight in a cool dark room. Next, the sections were incubated with a secondary antibody (biotin) that was reacted with an enzymatic agent (streptavidin). After a 5 min immersion in 3,3′ Diaminobenzidine (DAB) solution to allow for color development, the nuclei were stained and studied under a light microscope.

### 2.9. Statistical Analysis

Data are expressed as the mean ± standard error (SE). One-way analyses of variance followed by Bonferroni’s post-hoc tests were used to compare GFP expression after administration of GFP-MSCs. The Mann–Whitney U test was used to compare GFP expression between the siCXCR4/GFP-MSCs group and the GFP-MSCs group. Significance was defined at *p* < 0.05. Data analysis was performed using IBM SPSS Statistics 28.

## 3. Results and Discussion

### 3.1. CXCR4 Expression in Cultured MSCs

**Figure 1:**
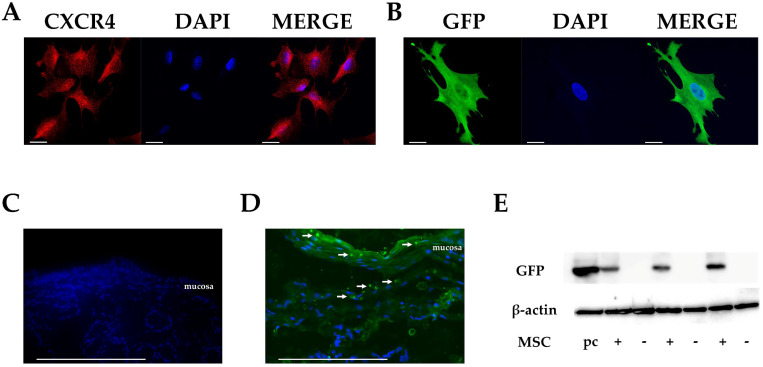
CXCR4 in cultured MSCs and the migration of MSCs into rectal tissues. (**A**,**B**) Immunocytochemical study of CXCR4 (red) and GFP (green) in cultured MSCs. Nuclei are stained blue by DAPI. Scale bar, 20 µm (×63). (**C**) Immunofluorescence (IF) staining of rectal tissue in TC (total control, rabbits not treated with CTX and to which MSCs were not administered) group (*n* = 3). GFP is indicated in green and nuclei in blue, as revealed by DAPI. No GFP-positive cells are found in the TC group. (**D**) IF staining of rectal tissue in IAG rabbits (treated with CTX and administered GFP-MSCs; intrarectal administration group). GFP is indicated in green and nuclei in blue, as revealed by DAPI (*n* = 3). GFP-MSCs (white arrows) are observed in the subepithelial mucosal and submucosal tissue as well as the surface of the rectal epithelium in IAG group. Scale bar, 200 µm (×40). (**E**) GFP expression in rectal tissues as revealed by Western blotting. Acronyms: pc, positive control; +, CTX and GFP-MSCs administered; -, control.

The clinical efficacy of MSCs depends on their ability to home to lesions. The chemokine/receptor axis is thought to play an important role in homing. Therefore, we first confirmed the expression of CXCR4, a chemokine receptor involved in homing, was maintained in cultured MSCs (Figure 1A). Next, to confirm the homing of intrarectally administered MSCs to the site of injury, we used cultured MSCs that were engineered to express GFP (Figure 1B).

### 3.2. Migration of MSCs in Rectal Tissues after Intrarectal Administration

To investigate the absorption of intrarectally administered MSCs into tissues in vivo, GFP fluorescence in rectal tissues was examined after administration. To study homing, we used a skeletal muscle injury model in which CTX was injected into the right vastus lateralis femoris of Japanese white rabbits. To ensure that MSCs home to the injury site, they were administered intrarectally one day after CTX administration, allowing time for the induction of inflammation and injury.

No GFP-positive cells were observed in the rectal tissues in the TC (total control) group (Figure 1C). Conversely, in the group that received intrarectal administration of GFP-MSCs (IAG group), GFP-positive cells were observed in the subepithelial mucosal and submucosal tissue, as well as the surface of the rectal epithelium 10 min after GFP-MSCs administration, as revealed by IF staining and WB (Figure 1D,E), confirming that intrarectally administered MSCs migrated into rectal tissues promptly after administration.

### 3.3. Homing of MSCs to Injury Sites after Intrarectal Administration

**Figure 2:**
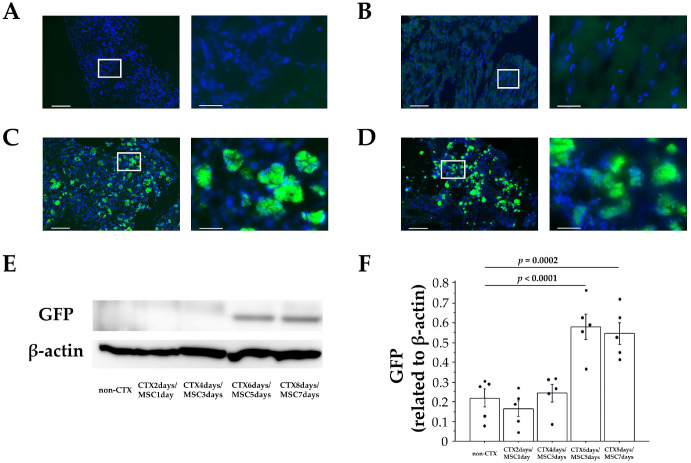
Immunofluorescent staining (IF) and Western blot analysis (WB) of GFP expression in injured skeletal muscle tissues. IF staining of the (**A**) non-CTX side, (**B**) CTX 6 days group, (**C**) CTX 6 days/MSCs 5 days group, and (**D**) CTX 8 days/MSCs 7 days group. GFP is indicated in green and nuclei in blue, as revealed by DAPI. Scale bars: **left**, 200 µm (×10); **right**, 40 µm (×60, Enlarged view of white frame). In the CTX 6 days/MSCs 5 days and CTX 8 days/MSCs 7 days groups, GFP-MSCs clusters were observed in the CTX-induced skeletal muscle lesion. (**E**) WB of GFP expression in injured tissues. (**F**) Quantification of GFP expression and its normalization by the expression of β-actin. GFP expression significantly increased in the CTX 6 days/MSCs 5 days and CTX 8 days/MSCs 7 days groups compared to the non-CTX treated side, CTX 2 days/MSCs 1 day group, and the CTX 4 days/MSCs 3 days group (*n* = 5 rabbits per group). Data represent the mean ± SE. *p* values were determined by one-way ANOVA.

Homing of MSCs after intrarectal administration was examined by analyzing GFP-positive cells and GFP expression (using IF and WB) at the site of skeletal muscle injury. GFP-positive cells were absent in the healthy skeletal muscle tissues of the non-CTX-treated side of the rabbits and the CTX-alone group (CTX 6 days) (Figure 2A,B). In the CTX 2 days/MSCs 1 day group and CTX 4 days/MSCs 3 days group, GFP-positive cells were absent at the injury site in all groups. However, in the CTX 6 days/MSCs 5 days group and CTX 8 days/MSCs 7 days group, GFP-positive cells were observed and converged into clusters at the injury site in all groups (Figure 2C,D). Moreover, WB showed significantly stronger GFP expression in the CTX 6 days/MSCs 5 days and CTX 8 days/MSCs 7 days groups compared with the non-CTX-treated side (*p* < 0.0001 and *p* = 0.0002, respectively) (Figure 2E,F). These results confirmed that intrarectally administered MSCs migrate to the site of injury within approximately five days. To our knowledge, this is the first evidence of the homing of intrarectally administered MSCs to lesions. These findings could be useful for future research and clinical applications involving intrarectal administration of cellular therapeutics.

### 3.4. CXCL12 and CXCR4 Expression in the Injury Area

**Figure 3:**
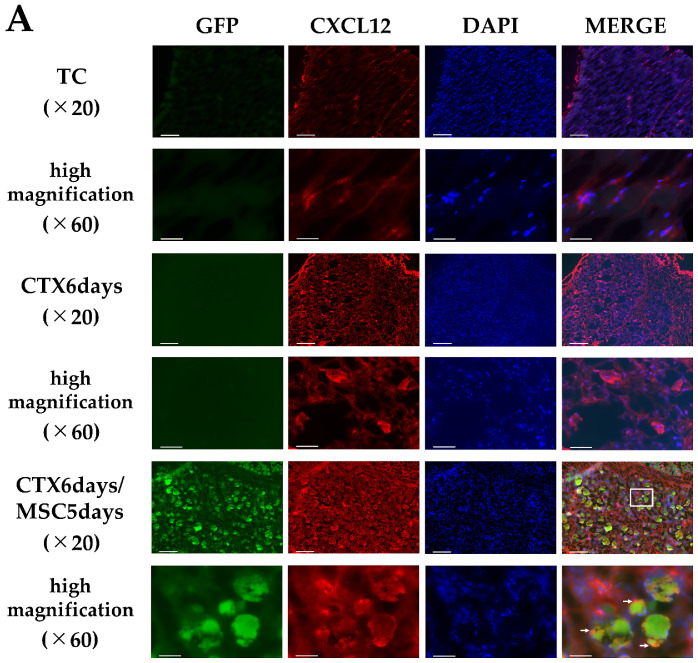

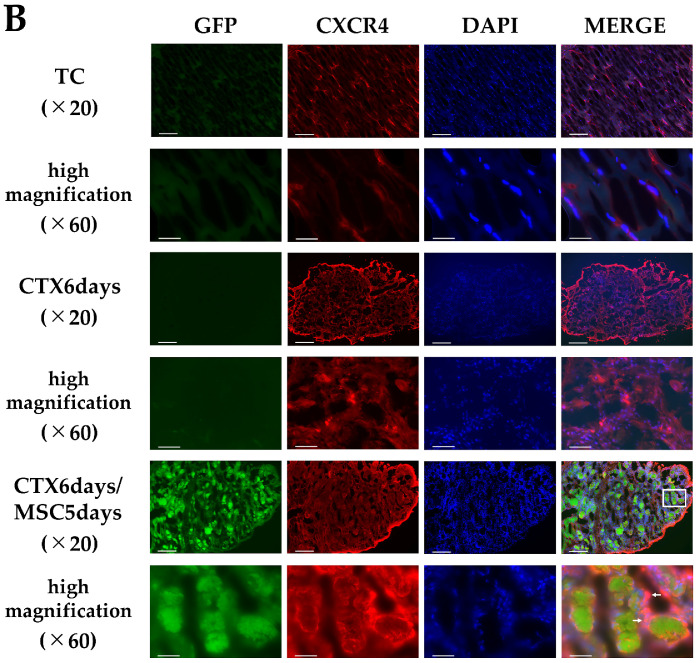
Immunofluorescent staining of (**A**) CXCL12 and (**B**) CXCR4 in rabbit skeletal muscle tissues. Representative immunofluorescent images of GFP (green), CXCL12 (red), and CXCR4 (red) in the TC (total control, rabbits not treated with CTX and to which MSCs were not administered) group (*n* = 3), CTX 6 days, and CTX 6 days/MSCs 5 days muscle tissues (*n* = 5 per group). In TC group, both CXCL12 and CXCR4 are marginally expressed. In the CTX 6 days group, CXCL12 and CXCR4 are expressed in the CTX-induced skeletal muscle inflammation/injury sites; increased expression was observed compared to that in the TC group. In the CTX 6 days/MSCs 5 days group, GFP-positive cells accumulated and clustered in the vicinity of the CXCL12-positive area (white arrows). Additionally, the CXCR4-positive cells and GFP-positive cells merged. At a high magnification, CXCR4 expression is observed on the surface layer of the GFP-positive cells (white arrows). White arrows indicate colocalization points between GFP and CXCR4. Scale bar: upper row, 200 µm (×10), lower row, 40 µm (×60) (Enlarged view of white frame).

Next, using IF staining, we evaluated the expression and localization of factors associated with MSC homing—CXCL12 and CXCR4—at the site of muscle injury. On the TC (total control) group, CXCL12 and CXCR4 were observed only sporadically. However, CXCL12 expression was observed in the muscle injury site in the CTX 6 days group. In the CTX 6 days/MSCs 5 days group, where MSCs homing was confirmed; staining for GFP and CXCL12 expression showed that GFP-MSCs were concentrated around CXCL12-positive areas. Similar to CXCL12, CXCR4 was expressed in the CTX 6 days/MSCs 5 days group. Since both MSCs and endogenous migratory cells express the homing factor CXCR4, CXCL12 could have induced the migration of endogenous CXCR4-positive cells other than the intrarectally administered MSCs to the site of muscle injury. Therefore, when we examined MSCs by staining GFP and CXCR4, some CXCR4-expressing cells merged with GFP-MSCs, confirming that CXCR4 was strongly expressed on the surface of MSCs (Figure 3).

### 3.5. Suppression of Homing by Knocking down CXCR4 in MSCs

**Figure 4:**
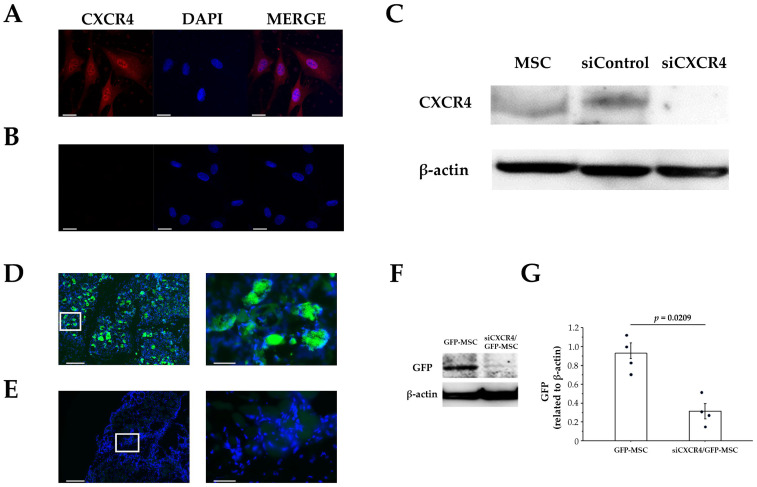
Suppression of homing by knocking down CXCR4 expression in MSCs. (**A**,**B**) Immunocytochemical study. CXCR4 is indicated in red and nuclei in blue, as revealed by DAPI. Scale bar, 20 µm (×63). (**A**) CXCR4 expression in MSCs in the absence of siRNA. (**B**) Knockdown of CXCR4 in MSCs by siRNA. (**C**) Western blot (WB) analysis of CXCR4 expression, showing downregulation of CXCR4 expression in MSCs by siRNA. (**D**,**E**) Immunofluorescent staining of GFP expression in injured skeletal muscle. GFP is indicated in green and nuclei in blue, as revealed by DAPI. Scale bars: **left**, 200 µm (×10); **right**, 40 µm (×60) (high magnification, Enlarged view of white frame). (**D**) 5 days after MSC administration. (**E**) siCXCR4/GFP-MSC administration. The appearance of GFP-positive cells is reduced owing to the knockdown of CXCR4. (**F**) WB analysis of GFP expression. (**G**) The expression of GFP was normalized to that of β-actin. The expression at the muscle injury site after knockdown of CXCR4 is significantly decreased. Each dot represents the data from an individual rabbit (*n* = 4 per group). Data represent the mean ± SE. *p* values were determined using the Mann–Whitney U test.

We confirmed the involvement of the CXCL12/CXCR4 axis in homing by using siCXCR4/GFP-MSCs generated by knocking down CXCR4 using siRNA (Figure 4A–C). These siCXCR4/GFP-MSCs were administered intrarectally to investigate homing to the site of skeletal muscle injury. Homing of siCXCR4/GFP-MSCs to the site of muscle injury was examined 5 days after MSC administration, since our earlier results showed that GFP-positive cells significantly increased at injury sites by day 5. The appearance of GFP-positive cells at the injury site was significantly lower (*p* = 0.0209) for intrarectally administered siCXCR4/GFP-MSCs than for intrarectally administered GFP-MSCs (Figure 4D–F), suggesting that the CXCL12/CXCR4 axis is involved in homing.

### 3.6. Staining of Nucleated Fibers

**Figure 5:**
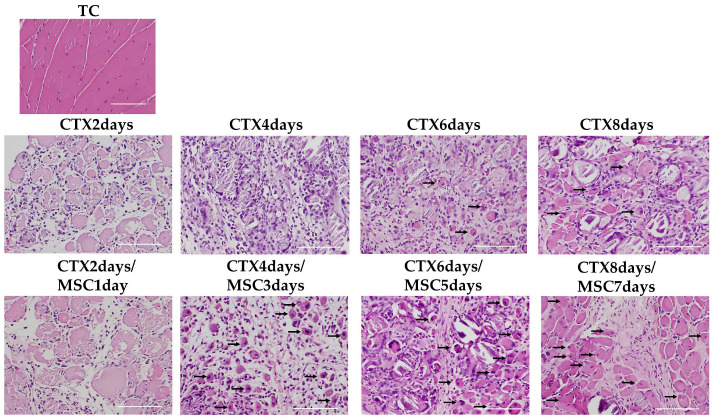
Hematoxylin and eosin staining in the skeletal muscle. TC is the total control group (rabbits not treated with CTX and to which MSCs were not administered). After CTX administration, skeletal muscle fiber necrosis and mononuclear cell infiltration peaked in the CTX 4 days group. Repair started in the CTX 6 days group, central nucleation occurred (arrows on representative parts), and interstitial fibrosis and mild regenerative changes in skeletal muscle fibers were observed (*n* = 3 per group). Meanwhile, in the MSCs administration group, central nucleation appeared in the CTX 4 days/MSCs 3 days group (arrows on representative parts), where the extent of skeletal muscle fiber necrosis was decreased and regeneration was promoted. In the CTX 6 days/MSCs 5 days and CTX 8 days/MSCs 7 days groups, there was a presence of increased central nucleation (arrows on representative parts) at the margin (*n* = 5 per group). Scale bar, 100 µm (×20).

H&E-stained specimens were examined to confirm the efficacy of rectally administered MSCs on the injury site. From 2 to 4 days after CTX injection, several inflammatory cells infiltrated the injured muscle in both groups. For the CTX-only administration groups, centrally nucleated fibers started to appear on day 6 (CTX 6 days). In contrast, in the MSCs-treated group, fibers with central nuclei began to appear on day 4 (CTX 4 days/MSCs 3 days) and increased on days 6 and 8 (CTX 6 days/MSCs 5 days, CTX 8 days/MSCs 7 days) (Figure 5). 

**Figure 6:**
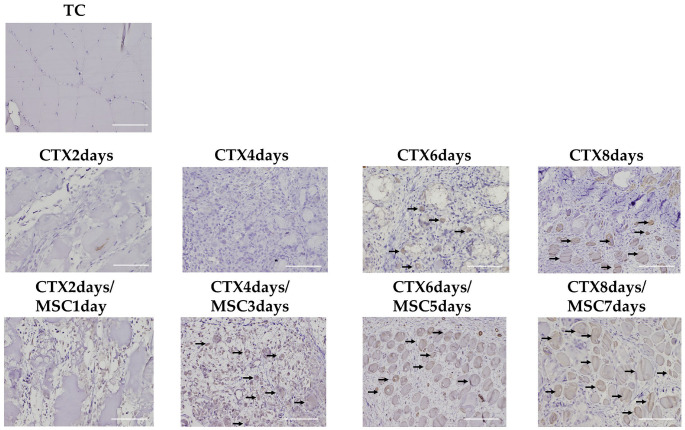
Immunohistochemical staining of Myh3 expression. Myh3 expression was not observed in the TC (total control, rabbits not treated with CTX and to which MSCs were not administered) group, while it was observed in the CTX 6 days group, increasing in the CTX 8 days group (arrows on representative parts) (*n* = 3 per group). In the MSCs administration group, Myh3 expression was observed in the CTX 4 days/MSCs 3 days group, with expression increasing gradually in the CTX 6 days/MSCs 5 days and CTX 8 days/MSCs 7 days groups, with the cell diameter also increasing (arrows on representative parts) at the margin (*n* = 5 per group). Scale bar, 100 µm (×20).

Next, we investigated Myh3, an indicator of early muscle regeneration [46,47], using IHC examination. No Myh3 expression was observed in the TC (total control), CTX 2 days, and CTX 2 days/MSCs 1 day groups. For the CTX-only administration groups, Myh3-positive cells began to appear in the CTX 6 days group. Meanwhile, for the MSCs administration groups, Myh3 expression was observed in the CTX 4 days/MSCs 3 days group. Additionally, compared to the CTX-only administration groups, the MSCs administration groups had more Myh3-expressing cells in the CTX 6 days/MSCs 5 days group and CTX 8 days/MSCs 7 days group (Figure 6). Histopathological examination and Myh3 expression analysis showed that intrarectal administration of MSCs promoted tissue repair and regeneration. It has also been reported that MSCs administered remotely have beneficial effects similar to endocrine actions [48,49]. In this study, the tissue repair tendencies and scattered regenerated skeletal muscles that began to appear in the CTX 4 days/MSCs 3 days group before homing was thought to be involved in endocrine action by the systemic administration of MSCs.

## 4. Conclusions

In this study, we investigated the feasibility of intrarectal administration of MSCs to find an administration route that is non-invasive and easier to administer than local implantation or intravenous administration by injection or surgery. The findings of this study contribute to future research on the clinical application of regenerative medicine. 

In this study, we focused on the CXCL12/CXCR4 axis as a factor involved in homing. CXCL12 expression increases at inflammation sites and in necrotic tissue. Recently, CXCL12 expression was shown to be elevated in patients with DMD and in mdx mice, which is known as a DMD model [50,51,52]. Elevation of CXCL12 expression in tissues suggests that the systemic administration of MSCs expressing CXCR4, which is a receptor for CXCL12, could be effective. Herein, we focused on the homing of MSCs from intrarectal administration to the lesion site; thus, we used a drug-induced local skeletal muscle injury model. In the future, it will be necessary for clinical applications to examine whether similar results can be obtained in the mdx mice, which is a severe and difficult-to-treat DMD disease model.

Although the homing of intrarectally administered MSCs was confirmed in this study, the homing rate was not examined. Recently, researchers have focused on the issue of low homing and decreased survival rates of MSCs post-transplantation [53,54]. Thus, they are attempting to promote MSCs homing and engraftment by enhancing expression of CXCR4 in MSCs [37,38,55], pretreating MSCs with inflammatory cytokines [16], and using MSCs spheroids [54]. Future studies are needed to improve the homing efficiency of intrarectally administered MSCs. 

This study confirmed that there was homing of MSCs to the lesion from intrarectal administration, which is a non-invasive and simple administration route. Intrarectal administration of MSCs may provide a route of administration for MSCs at home, which could be very useful for future stem cell research.

## Data Availability

The datasets used and/or analyzed during the current study are available from the corresponding author on reasonable request.

## Abbreviations

MSCs	Mesenchymal stem cells
CTX	cardiotoxin
CXCL12	C-X-C chemokine ligand 12
CXCR4	C-X-C chemokine receptor-4
Myh3	Myosin heavy polypeptide 3
DMD	Duchenne muscular dystrophy
SDF-1	stromal cell-derived factor-1
PDGFRα	Platelet-Derived Growth Factor Receptor α
mTORC1	mechanistic/mammalian target of rapamycin complex 1
GFP	Green Fluorescent Protein
IF	immunofluorescence
WB	Western blotting
IHC	immunohistochemical
PBS	phosphate-buffered saline
H & E	hematoxylin and eosin
DAPI	4′,6-diamidino-2-phenylindole
DAB	3,3′ Diaminobenzidine
